# Maturation of Complex Synaptic Connections of Layer 5 Cortical Axons in the Posterior Thalamic Nucleus Requires SNAP25

**DOI:** 10.1093/cercor/bhaa379

**Published:** 2020-12-26

**Authors:** Shuichi Hayashi, Anna Hoerder-Suabedissen, Emi Kiyokage, Catherine Maclachlan, Kazunori Toida, Graham Knott, Zoltán Molnár

**Affiliations:** 1 Department of Physiology, Anatomy and Genetics, University of Oxford, Oxford OX1 3PT, United Kingdom; 2 Department of Anatomy, Kawasaki Medical School, Kurashiki, Okayama 701-0192, Japan; 3 Department of Medical Technology, Kawasaki University of Medical Welfare, Kurashiki, Okayama 701-0193, Japan; 4 BioEM Facility, School of Life Sciences, EPFL, Lausanne 1015, Switzerland; 5 Research Center for Ultra-High Voltage Electron Microscopy, Osaka University, Ibaraki, Osaka 567-0047, Japan; 6 The Francis Crick Institute, London NW1 1AT, United Kingdom

**Keywords:** corticothalamic projections, giant bouton, layer 5, Rbp4, synapse

## Abstract

Synapses are able to form in the absence of neuronal activity, but how is their subsequent maturation affected in the absence of regulated vesicular release? We explored this question using 3D electron microscopy and immunoelectron microscopy analyses in the large, complex synapses formed between cortical sensory efferent axons and dendrites in the posterior thalamic nucleus. Using a Synaptosome-associated protein 25 conditional knockout (*Snap25* cKO), we found that during the first 2 postnatal weeks the axonal boutons emerge and increase in the size similar to the control animals. However, by P18, when an adult-like architecture should normally be established, axons were significantly smaller with 3D reconstructions, showing that each *Snap25* cKO bouton only forms a single synapse with the connecting dendritic shaft. No excrescences from the dendrites were formed, and none of the normally large glomerular axon endings were seen. These results show that activity mediated through regulated vesicular release from the presynaptic terminal is not necessary for the formation of synapses, but it is required for the maturation of the specialized synaptic structures between layer 5 corticothalamic projections in the posterior thalamic nucleus.

## Introduction

Synaptic development involves coordinated morphological changes of presynaptic and postsynaptic neurites such as formation of boutons and spines. Presynaptic mechanisms for those processes include neuronal activity and molecular signals that target postsynaptic dendrites (Shen and Cowan 2010; Bleckert and Wong 2011; Andreae and Burrone 2018; Favuzzi and Rico 2018). The role of neuronal activity in synapse formation in the brain has been extensively studied with different approaches. Blocking synaptic transmission by knocking out the gene for mammalian uncoordinated (Munc)-13, Munc-18, or the SNARE-complex protein Synaptosome-Associated Protein 25 (SNAP25) does not affect synapse formation in embryonic stages (Augustin et al. 1999; Verhage et al. 2000; Varoqueaux et al. 2002; Washbourne et al. 2002). More recent studies have supported the idea that synaptic transmission is dispensable for the formation and maintenance of synapses or spines in postnatal hippocampal CA1 by analyzing neurons with neonatal deletion of subunits of the NMDAR and AMPA receptors (Lu et al. 2013), organotypic slice cultures of double knockout of *Munc13-1* and *13-2* (Sigler et al. 2017), or Emx1-driven tetanus toxin (TeNT)-expressing brains (Sando et al. 2017). However, this only addresses the effect of silencing cortical neurons in the cortex and hippocampus. How does it affect synapse morphogenesis and maturation in regions that receive large and powerful cortical inputs such as the thalamic glomerular type connections in the thalamus (Hoogland et al. 1991; Groh et al. 2008; Hoerder-Suabedissen et al. 2018; Maclachlan et al. 2018)?

Corticofugal projections from cortical layer 5 provide major descending projections to subcortical areas, and those from the motor, sensory, and visual cortices have branches into “higher-order (associate)” thalamic nuclei (Deschenes et al. 1994; Kita and Kita 2012). It has been proposed that those layer 5 corticothalamic projections mediate transthalamic communication of different cortical areas (Sherman and Guillery 2011; Saalmann 2014). Layer 5 corticothalamic projections from somatosensory cortex in mice are known to have characteristic large boutons (giant boutons) in the posterior thalamic nucleus (Po), one of the higher-order thalamic nuclei (Hoogland et al. 1991; Groh et al. 2008; Hoerder-Suabedissen et al. 2018; Maclachlan et al. 2018). Unlike layer 6 corticothalamic projections, which have always small boutons, layer 5 corticothalamic projections from primary somatosensory (S1) cortex to Po provide powerful inputs to postsynaptic neurons mediated through ionotropic glutamate receptors (Reichova and Sherman 2004; Groh et al. 2008). This morphological and electrophysiological relationship suggests that the large bouton structure of layer 5 is essential for faithfully transferring powerful inputs to thalamic neurons (Sherman and Guillery 2011).

*Snap25* null mice show normal brain development during embryonic stages, but the neonatal lethality hampers the study on the role of SNAP25 in synaptic development during postnatal periods (Molnár et al. 2002; Washbourne et al. 2002). Our recent study using a cortical layer 5 Cre driver, *Rbp4-Cre*, and *Snap25* conditional knockout (*Snap25* cKO) mice has demonstrated that *Snap25* deletion in cortical layer 5 does not affect their axon projections and targeting during development but causes degeneration of their axons in the adult brain (Hoerder-Suabedissen et al. 2019). The utilization of *Rbp4-Cre*-driven *Snap25* cKO has some advantages to study the development of synapses in the corticothalamic system. First, *Rbp4-Cre* starts to be expressed at late embryonic stages (Grant et al. 2016), which is early enough to understand how layer 5 specialized giant boutons develop during the postnatal periods. Second, since *Snap25* removal does not affect the ingrowth of layer 5 corticothalamic projections into thalamic nuclei (Hoerder-Suabedissen et al. 2019), we can specifically address the effect of *Snap25* ablation on the specialized synaptic development. Therefore, using *Rbp4-Cre*-driven ablation of *Snap25* in this study, we found that layer 5 giant boutons, and their synapses in Po, develop during the second and third postnatal weeks. However, although presynaptic SNAP25 is dispensable for the initial formation of these synapses, their subsequent maturation, which includes the growth of boutons and protrusion of multiple excrescences from thalamic dendrites, appears to be impeded. Our results, therefore, suggest that the presynaptic control of regulated vesicular release by SNAP25 is essential for establishing the specialized synaptic connections of layer 5 corticothalamic projections.

## Materials and Methods

### Breeding and Maintenance of Transgenic Mice

The animal experiments were either performed in the Biomedical Services of the University of Oxford (UK) under an Animals (Scientific Procedures) Act 1986 project license as well as with local ethical approval by the Central Committee on Animal Care and Ethical Review (ACER) and the Animal Welfare and Ethical Review Body (AWERB) at the University of Oxford. Or they were performed at Kawasaki Medical School with the approval of the Animal Research Committee of Kawasaki Medical School. Tg(Rbp4-cre)KL100Gsat/Mmucd (Rbp4-Cre; Jackson Laboratories) mice were crossed with B6;129S6-Gt(ROSA)26Sortm14(CAG-tdTomato)Hze/J (Ai14) to label cortical layer 5 neurons. To generate *Rbp4-Cre*; *Snap25^fl/fl^* mice, the above strain was crossed with B6-Snap25tm3mcw (*Snap25^fl/fl^*) mice, which were obtained from University of New Mexico (Michael C. Wilson) (Hoerder-Suabedissen et al. 2019). We used both male and female mice for the experiments.

### Cre-Dependent Adeno-Associated Viral Injection to Somatosensory Cortex

To trace the axonal projections of Cre + L5 axons in Po thalamus, AAV2-CAG-Flex-ArchT-GFP (University of North Carolina Vector core) was injected in primary somatosensory cortex (S1) of *Rbp4-Cre;Ai14* young adult animals (6 weeks old). Mice were deeply anesthetized with isoflurane and placed in a stereotaxic frame. Following midline skin-incision, a craniotomy was performed over right-hand side S1 cortex (1.5 caudal, 2.75–2.8 lateral). Virus-filled pulled glass micropipettes were inserted into the brain to the required depth (0.6–0.7), and 200 nL of virus were slowly pressure ejected into the brain. Pipettes were retracted 5 min after the last ejection of virus, followed by postsurgery repair and recovery of the animal. After 3-week postinjection survival, animals were terminally anesthetized and perfusion fixed with 4% paraformaldehyde (PFA, Electron Microscopy Sciences, 15714) and 0.2% glutaraldehyde (Electron Microscopy Sciences, 16220) in phosphate buffer (PB) at pH 7.4 as described in Pre-embedding Immunoelectron Microscopy.

### Immunohistochemistry

Young animals at 1–4 weeks of age and 3- to 4-month old adults were perfusion fixed with 4% formaldehyde (Sigma-Aldrich, F8775) in 0.1 M PBS, and dissected brains were postfixed in the same solution for 24 h at 4 °C. Brains were sectioned coronally at 50 μm on a vibrating microtome (Leica, VT1000S). Sections were incubated in PBS containing 3% BSA (Sigma-Aldrich, A9647) (blocking solution) for 2 h, followed by incubation with guinea pig anti-Vesicular Glutamate Transporter 1 (VGluT1) antibody (1:2000, Millipore, AB5905) in the blocking solution for overnight at 4 °C. Staining was visualized by incubation with biotinylated anti-guinea pig secondary antibody (1:500, Vector Laboratories, BA7000) for 2 h and then with Alexa 488 (1:500, Invitrogen, S32354) or Cy5 (1:500, Invitrogen, SA1011) streptavidin-conjugated antibody for 2 h at room temperature (RT). Sections were counterstained with 4′,6-diamidino-2′-phenylindole dihydrochloride to visualize the nuclei. For microglia staining, sections were incubated with rabbit anti-Iba-1 antibody (1:1000, FUJIFILM Wako Chemicals, 019-19741) for overnight at 20 °C and then with FITC-conjugated donkey anti-rabbit secondary antibody (1:500, Jackson ImmunoResearch, 711–095–152) and Hoechst 33342 (0.4 μg/ml, Sigma-Aldrich, B2261) for 2 h at 20 °C.

### Serial Block-Face Scanning Electron Microscopy

Serial block-face scanning electron microscopy (SBEM) was performed as previously described (Maclachlan et al. 2018). Briefly, *Rbp4-Cre;Ai14;Snap25^+/+^* and *Rbp4-Cre;Ai14;Snap25^fl/fl^* brains at P18 were perfused with 0.1 M PB containing 2% PFA (Electron Microscopy Sciences, 15 714) and 2.5% glutaraldehyde (Electron Microscopy Sciences, 16 220), at pH 7.4, and postfixed at RT for 2 h. Dissected brains were vibratome sliced at 80 μm, and fluorescent and bright-field images of Po regions at various magnifications were collected using an epifluorescence microscope (Leica DMR) and confocal microscope (LSM710 Zeiss). The position of tdTom+ boutons was imaged along with major blood vessels as fiducial marks so that boutons in fluorescence images could be localized in electron micrographs by their location with respect to these other features. The sections were postfixed in 1.5% potassium ferrocyanide (Sigma-Aldrich, 14 459-95-1) and 2% osmium tetroxide mixed together (Electron Microscopy Sciences, 19110). They were then stained with 1% thiocarbohydrazide (Sigma-Aldrich, 101001342) followed by 2% osmium tetroxide and then stained overnight in 1% uranyl acetate (Electron Microscopy Sciences, 22 400). The final stain was at 50 °C, in a lead aspartate solution at pH 5, washed in water, and infiltrated with Durcupan resin (Electron Microscopy Sciences). The sections were mounted between glass microscope slides coated in a mold separating agent (Glorex Inspirations, Switzerland, 62407445) and the resin hardened at 65 °C for 24 h. The sample was imaged with a SEM microscope (Merlin, Zeiss NTS) fitted with the 3View cutting system (Gatan, Inc., Pleasanton, CA). The size of obtained stacks was as follows: xy, 40.38 μm × 39.77 μm (6228 × 6133 pixels); z, 8.23 μm (50-nm interval) for *Rbp4-Cre;Ai14;Snap25^+/+^* and xy, 39.96 μm × 39.66 μm (6163 × 6117 pixels); z, 11.82 μm (50-nm interval) for *Rbp4-Cre;Ai14;Snap25^fl/fl^*. The obtained image series was aligned using the alignment functions in the TrakEM2 plugin of FIJI (Cardona et al. 2012). SBEM images were correlated with fluorescence images using landmark blood vessels and cell bodies, and tdTom+ boutons were found in SBEM images. tdTom+ boutons and their connecting dendrites were segmented using TrakEM2, and 3D models were then exported into the Blender software (https://www.blender.org/). The bouton and the dendrite including excrescences were selected to create mesh models, and their volume and surface area were measured in Blender by using the volume and surface area measurement functions of the NeuroMorph tools (Jorstad et al. 2018). When there was more than one bouton derived from the same axon, only the first was included in the quantitative analyses and others were excluded.

### Post-embedding Immunoelectron Microscopy

Mice were perfused with PB containing 2% PFA and 2.5% glutaraldehyde at pH 7.4. Brains were coronally sectioned at 80 μm and the Po region was dissected out under a fluorescence microscope (MZFLIII, Leica). Tissue pieces containing Po were stained with 2% uranyl acetate (Agar Scientific, AGR1260A) in 0.1 M sodium acetate buffer (Sigma-Aldrich, S2889) for 45 min–1 h and dehydrated through a graded series of methyl alcohol (70%, 90%, and absolute) at −20 °C. The tissues were embedded in LR gold resin (Agar Scientific, AGR1284) containing 0.5% benzil (Agar Scientific, AGR1285) under ultraviolet light for 16–18 h at −20 °C. Ultrathin sections (70 nm) were prepared on an ultramicrotome (Leica Ultracut S) and mounted on 200 mesh nickel grids (Agar Scientific, AGG2200N) coated with formvar (TAAB, F145/025). For immunolabeling, sections were blocked with 1% chicken egg albumin (Sigma-Aldrich, A5503) in PBS and incubated with rabbit anti-red fluorescent protein (RFP) antibody (1:500, PM005, MBL international) for 2 h and then with 20 nm gold particle-conjugated goat anti-rabbit (1:50, BBI solutions, EM.GAR20) for 1 h. For detection of PSD95, anti-PSD95 antibody (1:100, Synaptic Systems, 124014) and 10 nm gold particle-conjugated goat anti-guinea pig antibody (1:50, BBI solutions, EM.GAG10) were used. For negative control sections, the primary antibody was omitted. The sections were postfixed with 1% glutaraldehyde for 10 min and lightly counterstained with 2% uranyl acetate and 2.77% lead citrate solution (Agar Scientific, AGR1210). The immunolabeled sections were examined on a JEOL 1010 transmission electron microscope (JEOL) fitted with an Orius digital camera (Gatan).

### Pre-embedding Immunoelectron Microscopy

Mice were perfused with PB containing 4% PFA and 0.2% glutaraldehyde at pH 7.4. The brains were cut at 50 μm thick, and freeze and thaw of the slices were performed in 30% sucrose twice. For detection of AAV-eGFP, the slices were blocked in 1% BSA and incubated with anti-GFP antibody (1:500, Invitrogen, A-11122) for 24 h at 4 °C followed by goat biotinylated anti-rabbit antibody (1:250, Vector Laboratories, BA-1000) for 2 h at RT. Signals were enhanced with the ABC method according to the manufacturer’s instruction (Vector Laboratories, PK-6100) and visualized with 3,3′-diaminobenzidine tetrahydrochloride (DAB, Sigma-Aldrich, D3939) for 5 min at RT. The slices were postfixed with 3% glutaraldehyde and then 1% osmium tetroxide in PB for 1 h at RT and stained with 2% uranyl acetate in H_2_O for 45 min–1 h at RT. The slices were dehydrated through a graded series of ethyl alcohol (30%, 50%, 70%, 80%, 90%, and absolute) at 4 °C and replaced with acetone followed by Spurr low viscosity embedding medium, whose formula is as follows: ERL4221 (Agar Scientific, AGR1047R, 4.1 g), DER 736 Diglycidylether of Polypropyleneglycol (Agar Scientific, AGR1072, 0.95 g), Nonenyl Succinic Anhydride (Agar Scientific, R1055, 5.9 g), and Dimethylaminoethanol (S1) (Agar Scientific, R1067, 0.1 mL). The slices were embedded between Aclar film (Agar Scientific, AGL4458) and the resin was hardened at 60 °C for 24 h. The 70-nm-thick ultrathin sections were prepared and imaged with the same ultramicrotome and electron microscopy as in post-embedding electron microscopy.

For detailed analysis of synaptic profiles, samples were prepared and examined as described previously (Kiyokage et al. 2017). Briefly, brains were perfused and sectioned as described above, and sections were stained with rabbit anti-RFP antibody (1:1000, a kind gift from T. Kaneko) and then with biotinylated goat anti-rabbit antibody (1:200, Vector Laboratories, BA-1000). Signals were enhanced with the ABC method followed by incubation in 0.05% DAB and 0.01% H_2_O_2_. After dehydration, the sections were flat-embedded in Epon-Araldite between a glass slide and a coverslip, both of which were precoated with a liquid-releasing agent (Electron Microscopy Sciences, 70880). Selected areas for EM observation were cut into 70-nm-thick serial sections with an ultramicrotome (Reichert-Nissei Ultra-Cuts, Leica) and examined with a transmission electron microscope (JEM-1400, JEOL).

### Image Analysis and Statistics

Image J (https://imagej.nih.gov/ij/) software was used to analyze fluorescent and electron microscopy images. For the quantification of the size of boutons in images obtained with laser scanning confocal microscopy, maximum intensity projection was applied to the z-stack fluorescent images (5 μm in total thickness) containing the whole volume of the bouton of interest, and the size of the bouton in the projected images was measured. For the quantification of bouton density, tdTom+ and VGluT1+ boutons in 450 μm^3^ (xy: 30 μm × 30 μm, z: 0.5 μm) were counted, and the number per 1 × 10^3^ μm^3^ was compared as the density between control and *Snap25* cKO. For the quantification of the number of microglia in Po, Iba-1-positive cells in 1.28 × 10^6^ μm^3^ (xy: 160 μm × 160 μm, z: 50 μm) were counted, and the number per 1 × 10^−3^ mm^3^ was compared as the density between control and *Snap25* cKO. For the quantification of the boutons in electron micrographs, we randomly selected immunogold-positive boutons in sections and measured the cross-sectional area, total area of excrescences contained in each bouton, number of synapses, and the length of synapses. Obtained data were analyzed by one-way analysis of variance followed by the post hoc test with Dunn’s multiple comparison test or Mann–Whitney *U* test using Prism 4 software (GraphPad). In the box-and-whiskers graph, horizontal lines above, inside, and below the box indicate maximum, median, and minimum values, respectively, and the top and bottom of the box indicate 75th and 25th percentiles, respectively.

## Results

### Layer 5 Giant Boutons in Posterior Thalamic Nucleus Develop in the Second and Third Postnatal Weeks

**Figure 1:**
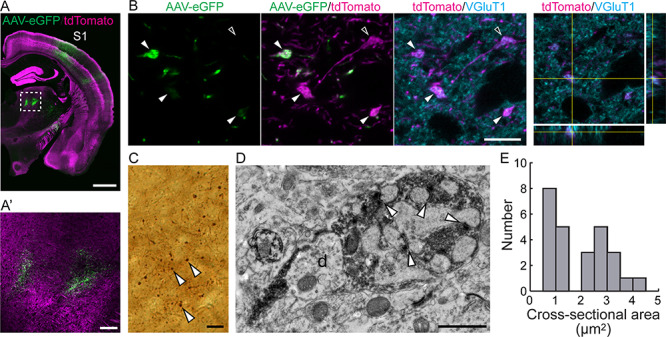
*Rbp4-Cre*+ layer 5 corticothalamic projections from S1 have giant boutons in adult Po at 9 weeks of age. (*A*, *B*) Laser scanning confocal microscopy images of an *Rbp4-Cre;Ai14* adult mouse brain injected with AAV-eGFP in S1 cortex. (*A*) Tiled image of *Rbp4-Cre*-dependent expression of AAV-eGFP injected into S1 cortex. (*A*′) Higher magnification of the boxed region in (*A*), which indicates *Rbp4-Cre*-driven eGFP+ (green) and tdTom+ (magenta) axon terminals in Po. (*B*) Typical examples of AAV-eGFP+ and/or tdTom+ boutons in Po, which are colocalized with the presynaptic marker VGluT1. The left 3 images show maximum intensity projections of part of a z-stack (1.0 μm out of 6 μm in total thickness) for each color shown in top. Orthogonal views at the point of one of the boutons shown in the right. Filled arrowheads and an open arrowhead indicate AAV-eGFP+;tdTom+ boutons and an AAV-eGFP-negative;tdTom+ bouton, respectively. (*C*) Light microscopy image of DAB-stained eGFP+ boutons in Po. Arrowheads indicate examples of eGFP+ boutons. (*D*) Pre-embedding immunoelectron microscopy against eGFP, with image taken in Po thalamus. White arrowheads point to synapses, “d” denotes dendrite. (*E*) Distribution histogram of the cross-sectional area of eGFP+ boutons in Po measured in electron micrographs. The presented net bouton area excludes the dendritic excrescences. *n* = 26 boutons from 3 brains were analyzed. Scale bars, 1 mm in (*A*), 100 μm in A′, 10 μm in (*B*), 20 μm in (*C*), 1 μm in (*D*).

Previous studies using injection of anterograde tracers, or Thy1 promoter-driven fluorescence labeling, have shown that layer 5 neurons from S1 cortex have giant boutons in Po of adult brains (Hoogland et al. 1991; Groh et al. 2008; Hoerder-Suabedissen et al. 2018). To study the synaptic development of layer 5 corticothalamic projections, we used the *Rbp4-Cre;Ai14* mouse line, whose tdTomato expression starts in a subset of layer 5 neurons at late embryonic stages (Grant et al. 2016; Hoerder-Suabedissen et al. 2019). This allows us to visualize their corticothalamic projections innervating Po in the early postnatal period. We first examined boutons that were positive for *Rbp4-Cre*-driven tdTomato fluorescence (tdTom+) in Po of *Rbp4-Cre*;*Ai14* adult brains. Although *Rbp4-Cre* is widely expressed in cortical layer 5, the somatosensory cortex including S1 is the major source of projections into Po (Allen Brain Connectivity Atlas). Consistent with this, injections of Cre-dependent AAV-eGFP virus to S1 cortex showed that eGFP-positive (eGFP+) axons project to Po (Fig. 1*A*), and they had large boutons, which colocalized with the presynaptic marker vesicular glutamate transporter 1 (VGluT1), in Po (Fig. 1*B*). Pre-embedding electron microscopy showed that eGFP+ boutons in Po contain multiple excrescences from Po dendrites (Fig. 1*C*,*D*). The cross-sectional area of boutons in randomly selected sections containing Po in adult brains was 2.0 ± 1.0 μm^2^ (mean ± SD, Fig. 1*E*, *n* = 26 boutons from 3 brains). The ultrastructural profile and the size of the boutons are comparable to those of *Rbp4-Cre*+ large boutons in Po in our previous study (Hoerder-Suabedissen et al. 2018). We previously reported that *Rbp4-Cre;Ai14* axons also form small boutons in Po and other thalamic nuclei (Hoerder-Suabedissen et al. 2018), and a recent study has also reported that *Rbp4-Cre+* layer 5 projections have large and small boutons (Prasad et al. 2020), but the small boutons were not the subject of these investigations, which specifically targeted the large boutons in Po with specialized synapses that could be easily identified with Cre-dependent viral tracing (AAV-eGFP) or with tdTomato (Fig. 1*A*,*B*). Our results suggest that the presynaptic bouton structure containing multiple dendritic excrescences is a general profile of *Rbp4-Cre*-positive large boutons in Po including those specifically derived from S1 cortex.

**Figure 2:**
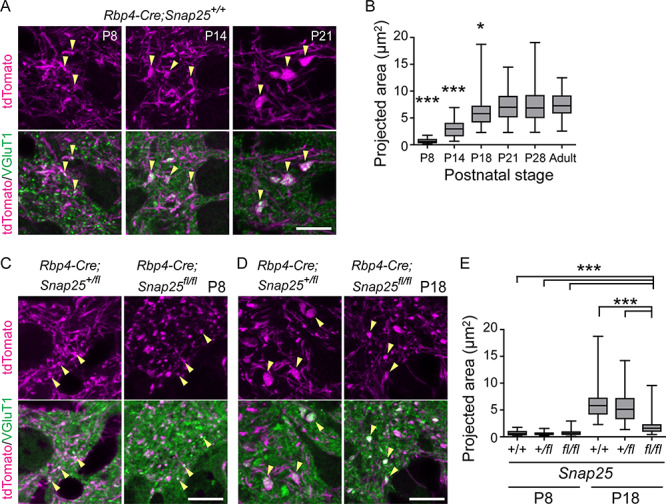
*Rbp4-Cre+* layer 5 boutons in Po during the first 4 postnatal weeks and in adult. (*A*) Laser scanning confocal microscopy images of *Rbp4-Cre+* layer 5 boutons in Po of *Rbp4-Cre;Ai14* mice at P8 (left), P14 (middle), and P21 (right). Maximum intensity projections of z-stack images (1.0 μm for P8 and P14, 2.5 μm for P21 in total thickness) are shown. Arrows indicate representative *Rbp4-Cre*-driven tdTomato+ boutons that are colocalized with VGluT1. (*B*) Quantification of the size of tdTom+ boutons in projected images of z-stacks in mice during postnatal development. The size of boutons at P8–P28 was compared with those of adults at 3–4 months of age. (*C*) tdTom+ boutons in *Rbp4-Cre;Snap25^+/fl^* and *Rbp4-Cre;Snap25^fl/fl^* at P8. (*D*) tdTom+ boutons in *Rbp4-Cre;Snap25^+/fl^* and *Rbp4-Cre;Snap25^fl/fl^* at P18. (*E*) Quantification of the size of tdTom+ boutons in projected images of z-stacks in *Snap25^+/+^*, *Snap25^+/f^*, and *Snap25^fl/fl^* at P8 and P18. There were no significant differences among the 3 genotypes at P8. The size of *Rbp4-Cre;Snap25^fl/fl^* boutons at P18 was significantly larger than those of the 3 genotypes at P8, but significantly smaller than those of controls at P18. *n* = 90 boutons from 3 brains for each genotype were analyzed in (*B*) and (*E*). ^*^P < 0.05; ^***^P < 0.001. One-way analysis of variance (ANOVA) followed by the post hoc test with Dunn’s multiple comparison test. Scale bars, 10 μm.

We next examined the development of synapses of tdTom+ axons in Po. Since it is difficult to label a particular population of *Rbp4-Cre+* layer 5 projections by viral injection in the perinatal cortex, we analyzed tdTom+ axons present in Po, which potentially contain not only axons from S1 but also those from outside S1. tdTom+ axons are shown to innervate Po in the first postnatal week (Grant et al. 2016; Hoerder-Suabedissen et al. 2019). tdTom+ boutons, which colocalized with VGluT1, were detected in Po at P8. To measure the size of boutons in fluorescent microscopy images, z-stack images covering the boutons of interest were projected onto a single plane with maximum intensity projection, and the area of the projected image (projected area) was analyzed. The size of the tdTom+ boutons increased by P21 (Fig. 2*A*,*B*). The size of the tdTom+ boutons at P21 and P28 was the same as that of the adult (Fig. 2*B*). These results indicate that the size of *Rbp4-Cre*-positive layer 5 boutons in Po increases during the second and third postnatal weeks.

### SNAP25 Is Required for the Normal Development of Layer 5 Boutons in Po

We next sought factors that regulate the development of layer 5 giant boutons in Po. We focused on SNAP25, which is essential for regulated vesicular release at synapses (Washbourne et al. 2002). We had previously validated the lack of regulated synaptic vesicle release in the floxed *Snap25* mouse model in the presence of Cre recombinase expression in vitro and in vivo (Marques-Smith et al. 2016; Hoerder-Suabedissen et al. 2019). This showed that there is no difference in the initial projections of *Rbp4-Cre*; *Snap25^fl/fl^* neurons into Po (Hoerder-Suabedissen et al. 2019). We found no difference in the size of the tdTom+ boutons at P8 between *Rbp4-Cre*; *Snap25^fl/fl^* and controls (*Rbp4-Cre*: *Snap25^+/+^* and *Rbp4-Cre*; *Snap25^+/fl^*) (Fig. 2*C*,*E*). At P18, however, the size of boutons in *Rbp4-Cre*; *Snap25^fl/fl^* was significantly smaller than that of controls (Fig. 2*D*,*E*), even though it was slightly, but significantly, increased compared with those of all the genotypes at P8. These results suggest that the growth of tdTom+ boutons in Po between P8 and P18 was impaired in *Rbp4-Cre*; *Snap25^fl/fl^*. We also compared the density of tdTom+ boutons between *Rbp4-Cre*; *Snap25^+/fl^* and *Rbp4-Cre*; *Snap25^fl/fl^* in Po. The number of tdTom+ and VGluT1+ boutons per 1 × 10^3^ μm^3^ in *Rbp4-Cre*; *Snap25^+/fl^* and *Rbp4-Cre*; *Snap25^fl/fl^* at P8 were 44.2 ± 13.3 and 52 ± 13.8, respectively (mean ± standard deviation [SD], *n* = 17 areas in Po from 3 brains), and there was no significant difference between the two (Supplementary Fig. 1, *P* = 0.64). In contrast, the bouton number in *Rbp4-Cre*; *Snap25^+/fl^* and *Rbp4-Cre*; *Snap25^fl/fl^* at P18 were 25.2 ± 9.1 and 43.9 ± 28.0, respectively (mean ± SD, *n* = 20 areas from 3 brains), and there was a significant difference between the two (Supplementary Fig. 1). The bouton density in *Rbp4-Cre*; *Snap25^+/fl^* at P18 was significantly decreased compared with that of the same genotype at P8, whereas that at P18 in *Rbp4-Cre*; *Snap25^fl/fl^* was less decreased and showed a wider distribution (*P* = 0.07). These results suggest that there is a refinement of boutons between P8 and P18 in control brains, but this refinement is impaired in *Snap25* cKO brains.

### Presynaptic SNAP25 Is Required for the Development of the Glomerular Type Junction between Layer 5 Boutons and Po Dendrites

**Figure 3:**
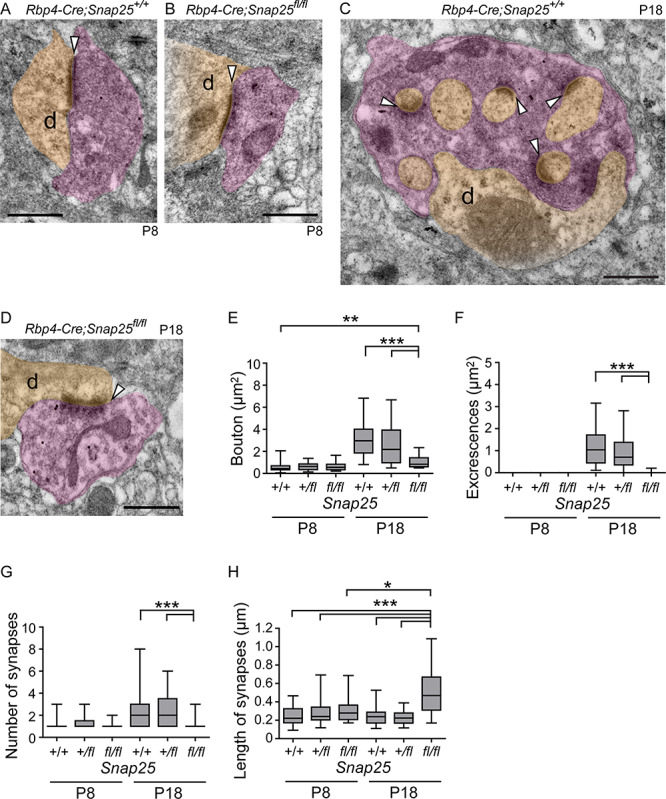
*Rbp4-Cre;Snap25^fl/fl^* boutons lack excrescences from Po dendrites at P18. (*A*, *B*) Ultrastructure obtained by post-immunoelectron microscopy of *Rbp4-Cre;Snap25^+/+^* (*A*) and *Rbp4-Cre;Snap25^fl/fl^* (*B*) synapses at P8, which do not contain any excrescences from the associated postsynaptic compartment. (*C*, *D*) Ultrastructure of *Rbp4-Cre;Snap25^+/+^* and *Rbp4-Cre;Snap25^fl/fl^* at P18. (*E*–*G*) Quantitative analysis of the size of boutons (*E*), the area of excrescences (*F*), and the number of synapses (*G*). Those parameters in *Snap25^fl/fl^* at P18 were significantly smaller than those of controls at P18. The size of boutons in *Snap25^fl/fl^* at P18 was significantly increased compared with that of *Snap25^+/+^* at P8 but not significantly different from those of *Snap25^+/f^* and *Snap25^fl/fl^* at P8 (*E*). (*H*) The length of synapses in each genotype at P8 and P18. The length of synapses in *Snap25^fl/fl^* at P18 was significantly larger than those of all the genotypes at P8 and those of controls at P18. Purple and light orange indicate presynaptic boutons and postsynaptic dendrites, respectively; “d” denotes the dendrite. Synapses with clear postsynaptic densities are indicated with white arrowheads. *n* = 30 boutons from 3 brains for each genotype were analyzed in (*E*–*H*). One-way ANOVA followed by the post hoc test with Dunn’s multiple comparison test. ^*^P < 0.05; ^**^P < 0.01; ^***^P < 0.001. Scale bars, 500 nm.

To understand what this size difference means in terms of their structure, we performed ultrastructural analysis of knockout boutons using post-embedding immunoelectron microscopy to identify those with tdTomato. At P8, tdTom+ boutons formed a synapse with Po dendrites in randomly selected EM sections of both control and *Rbp4-Cre*; *Snap25^fl/fl^* mice (Fig. 3*A*). At this age, there were no significant differences in the bouton size or the number of synapses per bouton in control and *Rbp4-Cre*; *Snap25^fl/fl^* mice (Fig. 3*A*,*B*,*E*,*G*). At P18, however, excrescences of Po dendrites showed multiple synapses with tdTom+ boutons in *Rbp4-Cre*; *Snap25^+/+^* brains (Fig. 3*C*). We also tried to find small boutons, which were reported in adult Po (Prasad et al. 2020) and form a single synapse with Po dendrites (Hoerder-Suabedissen et al. 2018), but we found no such tdTom+ small boutons in our EM sections of brains at P18. *Rbp4-Cre*; *Snap25^fl/fl^* boutons at P18 did not envelop excrescences and only showed a single synapse (Fig. 3*D*). The size of boutons, the area of excrescences, and the number of synapses per bouton, per section, were significantly smaller in *Rbp4-Cre*; *Snap25^fl/fl^* compared with controls (Fig. 3*E*–*G*, *n* = 30 boutons from 3 brains for each genotype per age). We also compared the size of synapses (length of synapses in sections) among the genotypes at P8 and P18 and found that it was significantly increased in *Rbp4-Cre*; *Snap25^fl/fl^* at P18 compared with those of controls at P18 as well as those of all the genotypes at P8 (Fig. 3*H*). These results indicate that SNAP25 in presynaptic neurons is required for the maturation of the excrescences on the dendrites of Po neurons and the subsequent formation of multiple synapses. Our previous study has shown that tdTom+ axons start to degenerate in juvenile *Rbp4-Cre*; *Snap25^fl/fl^* and degenerating boutons have dark cytoplasm and multivesicular bodies (Hoerder-Suabedissen et al. 2019). We did not find such cytoplasmic alteration in tdTom+ boutons at P8 or P18. Moreover, the number of microglia, which were known to increase in degenerating brain areas (Gomez-Nicola et al. 2013; Olmos-Alonso et al. 2016), was not different between *Rbp4-Cre*; *Snap25^+/fl^* and *Rbp4-Cre*; *Snap25^fl/fl^* in Po at P21 (Supplementary Fig. 2A,B). Those results suggest that the degenerative phenotype is not prominent in Po by the end of the third postnatal week.

**Figure 4:**
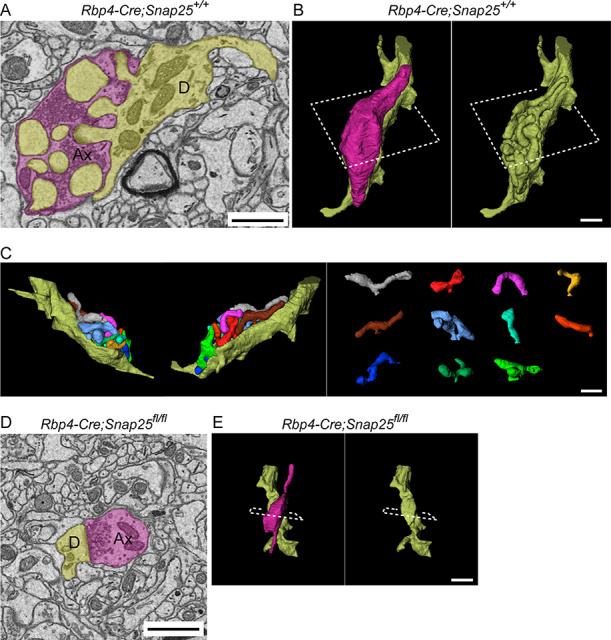
SBEM shows the structure of *Rbp4-Cre;Snap25^+/+^* and *Rbp4-Cre;Snap25^fl/fl^* boutons and their connected dendrites at P18. A single image from the series taken with the SBEM (*A*) and reconstructed 3D model (*B*) from Po in a *Rbp4-Cre;Snap25^+/+^* brain (Ax, axon; D, dendrite). (*C*) Reconstruction of individual excrescences on the same dendrite shown in (*A*, *B*). Each excrescence is shown with different color in the right panel. A typical SBEM image (*D*) and reconstructed 3D model (*E*) from Po in an *Rbp4-Cre;Snap25^fl/fl^* brain. Scale bars, 1 μm.

To better understand the effect that SNAP25 removal has on the structure of these junctions between layer 5 boutons and Po dendrites, we used correlative light and Serial block-face scanning electron microscopy (SBEM); reconstructing in 3D the boutons and excrescences at P18. Each *Rbp4-Cre*; *Snap25^+/+^* bouton contained multiple excrescences to which it was synapsing, all of which were derived from a single dendrite. The bouton completely enveloped the excrescences and glial processes covered the boutons (Fig. 4*A*,*B*, Supplementary Video 1). Reconstruction of individual excrescences showed their diverse morphology such as straight or curved shape with or without branching (Fig. 4*C*). Each process, or spine, of the excrescence is packed into a single bouton, each being independently isolated within the bouton, and unopposed to any other process of the same excrescence. In contrast, *Rbp4-Cre*; *Snap25^fl/fl^* boutons did not contain dendritic excrescences and had a single synapse with the shaft of the connecting Po dendrite (Fig. 4*D*,*E*, Supplementary Video 2). In 4 out of 11 boutons (axons) analyzed in *Rbp4-Cre*; *Snap25^fl/fl^*, single boutons formed 2 synapses with different dendrites, or axons formed multiple boutons within a short distance (~10 μm), each of which synapsed on different dendrites (Supplementary Fig. 3). No such axon that synapses on different dendrites within a short distance or within single boutons was found in *Rbp4-Cre*; *Snap25^+/+^*. *Rbp4-Cre*; *Snap25^+/+^* boutons contained 6.1 ± 2.8 (mean ± SD, *n* = 11 boutons from 1 brain) excrescences, whereas *Rbp4-Cre*; *Snap25^fl/fl^* boutons contained no excrescences (mean ± SD, *n* = 11 boutons from 1 brain, Table 1). The surface of boutons in contact with the dendrite was greatly reduced in *Rbp4-Cre*; *Snap25^fl/fl^* (1.1 ± 1.4 μm^2^, mean ± SD, Table 1) compared with that in *Rbp4-Cre*; *Snap25^+/+^* (41.5 ± 12.7 μm^2^ mean ± SD, Table 1). The total and net volume of boutons with and without including excrescences, in *Rbp4-Cre*; *Snap25^+/+^*, were 9.2 ± 2.3 μm^3^ and 5.7 ± 1.5 μm^3^, respectively (mean ± SD, *n* = 11 boutons from 1 brain, Table 1). Both of those volumes are larger than the volumes in *Rbp4-Cre*; *Snap25^fl/fl^* (2.2 ± 1.4 μm^3^ mean ± SD, *n* = 11 boutons from 1 brain, Table 1). Those results suggest that loss of excrescences in the *Rbp4-Cre*; *Snap25^fl/fl^* boutons results in reduction of the contact surface area and the number of synapses between the boutons and Po dendrites.

**Table 1:** Comparison of tdTom+ boutons and their connecting Po dendrites between *Rbp4-Cre*; *Snap25*^+/+^ and *Rbp4-Cre*; *Snap25^fl/fl^* in Po at P18

	1. Area of contact with dendrite per bouton (μm^2^)	2. Number of excrescences per bouton	3. Volume of excrescences per bouton (μm^3^)	4. Volume of bouton (μm^3^)	5. Net volume of bouton (μm^3^)^a^	6. Number of boutons analyzed
*Rbp4-Cre*; *Snap25*^+/+^	41.5 ± 12.7	6.1 ± 2.8	3.5 ± 0.9	9.2 ± 2.3	5.7 ± 1.5	11
*Rbp4-Cre*; *Snap25^fl/fl^*	1.1 ± 1.4	0	0	2.2 ± 1.4	2.2 ± 1.4	11

^a^(5) = (4)–(3)

### SNAP25 Is Required for the Formation of Normal Synaptic Structures

**Figure 5:**
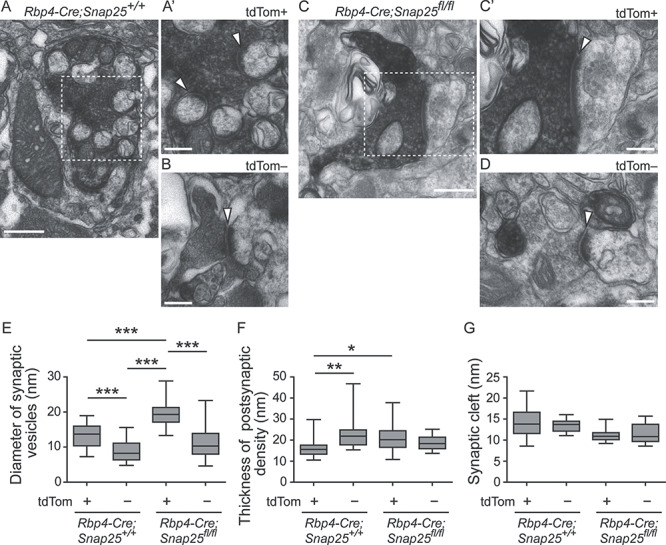
Assembly of synapses between *Rbp4-Cre+* layer 5 boutons and Po dendrites in control and *Rbp4-Cre;Snap25^fl/fl^* brains at P18. (*A*, *B*) Electron micrographs obtained by pre-embedding immunoelectron microscopy against tdTomato showing synapses formed by *Rbp4-Cre*-driven tdTomato-positive (tdTom+) (*A*, *A*′) or tdTom− boutons (*B*) with Po dendrites in *Rbp4-Cre;Snap25^+/+^* brains. (*A*′) A magnified image of the boxed region in (*A*). Arrowheads indicate synapses. (*C*, *D*) Electron micrographs showing synapses formed by tdTom+ (*C*, *C*′) or tdTom− boutons (*D*) with Po dendrites in *Rbp4-Cre;Snap25^fl/fl^* brains. (*C*′) A magnified image of the boxed region in (*C*). Arrowheads indicate synapses. (*E*–*G*) The diameter of synaptic vesicles in presynapses (*E*), the thickness of postsynaptic density (*F*), and the distance of synaptic cleft (*G*) of tdTom+ and tdTom− synapses in *Rbp4-Cre;Snap25^+/+^* and *Rbp4-Cre;Snap25^fl/fl^* brains. *n* = 53, 41, 67, and 42 vesicles from the left in (*E*) and *n* = 17, 13, 23, and 17 synapses from the left in (*F*) and (*G*), obtained from one brain for each genotype. One-way ANOVA followed by the post hoc test with Dunn’s multiple comparison test. ^*^P < 0.05; ^**^P < 0.01; ^***^P < 0.001. Scale bars: 500 nm in (*A*) and (*C*) and 200 nm in (*A*′), (*B*), (*C*′), and (*D*).

We compared the ultrastructure of synapses in Po between *Rbp4-Cre*; *Snap25^+/+^* and *Rbp4-Cre*; *Snap25^fl/fl^* at P18 by pre-embedding immunoelectron microscopy. In these analyses, we also compared tdTom+ boutons with tdTom-negative (tdTom−) boutons to find any difference in the synaptic structure between the two. In *Rbp4-Cre*; *Snap25^+/+^* brains, synaptic vesicles in tdTom+ giant boutons were round without dense core (Fig. 5*A*,*A*′). The diameters of those vesicles were significantly larger than those of asymmetrical synapses formed by tdTom− small boutons, which were found near the tdTom+ giant boutons analyzed (Fig. 5*B*,*E*, left 2 plots, *n* = 53 and 41 vesicles in tdTom+ and tdTom− presynapses, respectively, from 1 brain). In contrast, the thickness of postsynaptic densities in tdTom+ synapses was significantly smaller than those in tdTom− synapses (Fig. 5*F*, left 2 plots, *n* = 17 tdTom+ and 13 tdTom− synapses from 1 brain). The distance of their synaptic clefts showed no significant difference between the two (Fig. 5*G*, left 2 plots). These results suggest that tdTom+ giant boutons have a different synaptic profile from tdTom− small boutons in the size of vesicles and postsynaptic densities. In *Rbp4-Cre*; *Snap25^fl/fl^* brains, tdTom+ (*Snap25* cKO) presynapses also contained round vesicles without dense core (Fig. 5*C*,*C′*), and those vesicles were significantly larger than those of asymmetrical synapses formed by tdTom− small boutons (Fig. 5*D*,*E*, right 2 plots, *n* = 67 and 42 vesicles in tdTom+ and tdTom− presynapses, respectively, from 1 brain). Those vesicles in tdTom+ presynapses in *Rbp4-Cre*; *Snap25^fl/fl^* were even larger than the counterparts in *Rbp4-Cre*; *Snap25^+/+^* (Fig. 5*E*). Postsynaptic densities of tdTom+ synapses in *Rbp4-Cre*; *Snap25^fl/fl^* were also significantly larger than the counterparts in *Rbp4-Cre*; *Snap25^+/+^* (Fig. 5*F*, *n* = 17 and 23 synapses, respectively, in *Rbp4-Cre*; *Snap25^+/+^* and *Rbp4-Cre*; *Snap25^fl/fl^*), whereas there was no difference between the two in the distance of their synaptic clefts (Fig. 5*G*, first and third plots from the left). In all the parameters, there was no difference between tdTom− boutons in *Rbp4-Cre*; *Snap25^+/+^* and *Rbp4-Cre*; *Snap25^fl/fl^*, suggesting that *Rbp4-Cre*-driven *Snap25* removal specifically affected tdTom+ (*Rbp4*-Cre+) boutons. These results show that deletion of pre-synaptic SNAP25 also alters, as well as changing the bouton morphology, some of the synaptic profiles of the *Rbp4-Cre+* layer 5 giant boutons.

**Figure 6:**
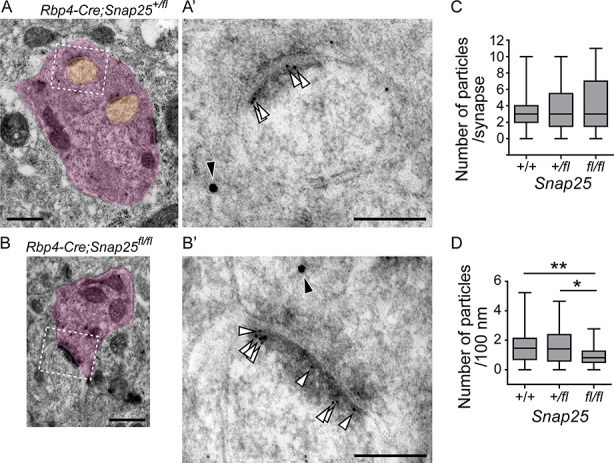
Postsynaptic localization of PSD95 at synapses between layer 5 boutons and Po dendrites in control and *Rbp4-Cre;Snap25^fl/fl^* brains at P18. (*A*, *B*) Ultrastructure of an *Rbp4-Cre+* layer 5 bouton and its connecting dendrite in *Rbp4-Cre;Snap25^+/fl^* (*A*) and *Rbp4-Cre;Snap25^fl/fl^* (*B*). 10 and 20 nm immunogold particles were used to detect PSD95 and tdTomato, respectively, by post-embedding immunoelectron microscopy. (*A*′, *B*′) Magnified images of the boxed region in (*A*) and (*B*), respectively. White and black arrowheads indicate 10 and 20 nm immunogold localization, at postsynaptic density. (*C*, *D*) The number of immunogold particles per synapse (*C*) and the number of particles per 100 nm (*D*) were compared in the 3 genotypes. *n* = 52, 50, and 34 synapses from 3 brains for each of *Rbp4-Cre;Snap25^+/+^, Rbp4-Cre;Snap25^+/fl^,* and *Rbp4-Cre;Snap25^fl/fl^*. One-way ANOVA followed by the post hoc test with Dunn’s multiple comparison test. ^*^P < 0.05; ^**^P < 0.01. Scale bars, 500 nm in (*A*) and (*B*), 200 nm in (*A*′) and (*B*′).

We also compared the localization of postsynaptic density protein (PSD) 95 as a marker of postsynaptic densities by post-embedding immunoelectron microscopy of tdTom+ boutons in control animals (*Rbp4-Cre*; *Snap25^+/+^* and *Rbp4-Cre*; *Snap25^+/fl^*) and *Rbp4-Cre*; *Snap25^fl/fl^*. PSD95 localized at postsynaptic densities on excrescences of Po dendrites that synapse with tdTom+ boutons in control at P18 (Fig. 6*A*,*A*′). Postsynaptic densities on Po dendrites that synapse with tdTom+ (*Snap25* cKO) boutons in *Rbp4-Cre*; *Snap25^fl/fl^* brains also contained PSD95 (Fig. 6*B*,B′). The number of immunogold particles per synapse was not significantly different between control and *Rbp4-Cre*; *Snap25^fl/fl^* (Fig. 6*C*). However, since the length of synapses was significantly larger in *Rbp4-Cre*; *Snap25^fl/fl^* than in control (Fig. 2*H*), the density of particles (the number of particles per 100 nm) in *Rbp4-Cre*; *Snap25^fl/fl^* was significantly decreased compared with that in control (Fig. 6*D*). These results support the idea that assembly of synapse structure itself is not much affected, but some synaptic profiles are altered by SNAP25 removal from presynapses of *Rbp4-Cre*+ layer 5 giant boutons.

## Discussion

We show that the maturation of specialized junctions between cortical layer 5 axons from somatosensory cortex and Po thalamic dendrites requires the presence of SNAP25. Morphologically, the initial synapse formation of *Rbp4-Cre*-positive layer 5 corticothalamic projections in Po does not appear to be affected by the absence of SNAP25. However, their subsequent maturation, with the formation of excrescences of Po dendrites, envelopment by the bouton to form a single glomerulus is suppressed. These results suggest that SNAP25-mediated regulated vesicular release from presynaptic terminals plays a crucial role in coordinated morphogenesis of the pre- and postsynaptic structure of layer 5 corticothalamic projections in Po.

### Ultrastructure of the Interface between Layer 5 Boutons and Po Dendrites

By using immunoelectron microscopy, we have shown that the synaptic development of layer 5 corticothalamic projections in Po takes 2 steps: the axons form small boutons that synapse on the shaft of Po dendrites by the end of the first postnatal week and then the boutons become larger in the second and third postnatal weeks. Each of those large boutons contains approximately 10 excrescences from Po dendrites. Mossy fibers from the hippocampal dentate gyrus also form characteristic large boutons that synapse with thorny spines on CA3 dendrites (Rollenhagen and Lubke 2010). The mossy fiber boutons have 2–13 μm^3^ in volume, and this size is comparable to that of layer 5 boutons in Po. Both boutons contain numerous mitochondria. However, there are also differences between the 2 giant boutons: the mossy fiber giant boutons have filopodial extensions that form contacts with GABAergic interneurons and CA3 dendrites (Acsády et al. 1998; Wilke et al. 2013; Martin et al. 2017), whereas no such extensions were observed in layer 5 giant boutons in Po. In CA3 thorny excrescences, multiple large spine heads often emerge from a common stalk (Wilke et al. 2013), whereas in Po dendrites, each excrescence has a long, slender shape with less branches. Compared with the mossy fiber boutons, the layer 5 boutons appear to wrap all the connecting excrescences more completely and glial processes further cover the bouton.

*Snap25* wild-type boutons have an area of contact with the connecting Po dendrites that is 35 times larger than its knockout counterpart. This shows a massive expansion of the contact surface up to the P18 time point with the formation of the excrescence structures. Each bouton forms multiple synapses with excrescences in *Snap25* wild type at P18, and the size of those synapses was not different from those of single synapses formed at P8. In contrast, in *Snap25* cKO, the number of synapses at P18 was unchanged from P8 due to the failure of the excrescence formation, but the size of the single synapses became larger at P18 than those at P8. Although it is not clear how this is caused, one possibility is that synaptic expansion is induced by an adaptation to inactivity, which is well studied in cultured hippocampal neurons (Murthy et al. 2001). It is also possible that the slight growth of the *Snap25* cKO boutons between P8 and P18 may increase contact surface between layer 5 boutons with Po dendrites and induces the increase of their synaptic size. This synapse expansion in *Snap25* cKO does not appear to accompany an increase of PSD95 assembly as shown by the reduced density of PSD95 signals in *Snap25* cKO synapses.

### Differential Effects of Presynaptic Silencing on Postsynaptic Dendritic Structures

Our results have shown that the initial synaptic assembly is not affected by *Rbp4-Cre*; *Snap25^fl/fl^*. This suggests that synaptic communication via SNAP25 is dispensable for the assembly of the initial synapses but essential for the subsequent establishment of specialized synaptic structure with the protrusion of dendritic excrescences and layer 5 bouton invaginations. Importantly, in the *Rbp4-Cre*; *Snap25^fl/fl^* brains we used, *Snap25* was removed only in presynapses of layer 5 giant boutons in Po, not the Po neurons themselves. Lack of excrescences on Po dendrites in those brains therefore suggests that presynaptic vesicular release is essential for the morphological changes of postsynaptic structures in Po neurons. Normal assembly of synapses under silencing is in agreement with previous observations obtained by other silencing methods in the hippocampus (Lu et al. 2013; Sando et al. 2017; Sigler et al. 2017). This suggests that the initial assembly of synapses is regulated by release-independent mechanisms such as synaptic organizing cell adhesion molecules (Siddiqui and Craig 2011), but the subsequent maturation and/or further morphological changes of synaptic contact sites such as formation of excrescences require evoked synaptic vesicle release or vesicle secretion from presynapses. However, the previous studies reported that there is a reduction in the number of synapses in the cortex of *Munc18-1* null mutants (Bouwman et al. 2004) and also that Emx1-driven TeNT causes a significant decrease of dendritic arborization and approximately 40% decrease of spine densities in the CA1 (Sando et al. 2017). Moreover, a study using live imaging of hippocampal organotypic slice culture showed that blocking postsynaptic NMDA and AMPA receptors with antagonists reduces recruitment of PSD95 to newly formed spines and the stability of spines (De Roo et al. 2008). Decreased recruitment of PSD95 was also observed in retinal ganglion cells (RGCs) in the synapse formed by bipolar cells that express TeNT (Kerschensteiner et al. 2009). Those results suggest that synaptic transmission may be involved in stabilization of newly formed spines, but the impact of silencing can be varied depending on the neuronal circuit analyzed and the method of perturbing synaptic communication.

### Developmental Regulation of Giant Bouton Formation

Multiple synaptic contacts between single presynaptic boutons and dendrites are also found in other thalamic nuclei including dorsal lateral geniculate nucleus (dLGN), in which axons from RGCs form large boutons (Guillery 1969; Bickford 2015; Morgan et al. 2016). In the dLGN, the bouton size is initially small at P7 and large boutons emerge by P14 (Bickford et al. 2010). This timing of giant bouton formation in dLGN is similar to that of layer 5 boutons in Po. In mossy fiber boutons in hippocampal CA3, synapses and excrescences also develop between P7 and P14, which increases the complexity of the pre- and postsynaptic structures (Wilke et al. 2013). The regulatory mechanism for the development of those large boutons is not fully understood. In hippocampal mossy fibers, postsynaptic ligand of Numb protein X 1 (Lnx1) and EphB receptors retrogradely regulate the fiber terminal maturation (Liu et al. 2018). It has also been shown that the interaction between the heparan sulfate proteoglycan GPC4 and the orphan receptor GPR158 organize mossy fiber-CA3 synapses (Condomitti et al. 2018). Are those molecular pathways also involved in the formation of the L5-Po synapses? It would be of interest to address this question by determining whether synaptic vesicular release via SNAP25 regulates the molecular pathways involved in the mossy fiber bouton formation. However, it could also be possible that the mechanism underlying postsynaptic morphological changes is different between mossy fiber-CA3 and layer 5-Po synapses. A previous study reported that the density of thorny spines is 30% increased, rather than decreased, compared with control by Emx1-driven TeNT expression (Sando et al. 2017), which cleaves members of the synaptobrevin/vesicle-associated membrane proteins family and suppresses synaptic vesicular release as *Snap25* knockout does. In *Gpr158* knockout mice, although the size of each mossy fiber bouton is decreased and synaptic architecture is impaired, the density of their presynaptic terminals and thorny excrescences is increased (Condomitti et al. 2018). These results suggest that different molecular pathways operate in parallel to control the formation and morphological maturation of boutons and excrescences as well as synaptic development. Comparing the effect of silencing of presynaptic neurons with the same method between mossy fiber-CA3 and layer 5-Po systems would provide further insights into the underlying mechanism for the specialized synaptic structures in the 2 systems.

Previous studies have revealed electrophysiological properties of layer 5-Po synapses (Reichova and Sherman 2004; Groh et al. 2008) and those with anatomical evidence provide an important framework for the function of the cortico-thalamo-cortical loop (Sherman and Guillery 2011). *Snap25* cKO can be a good model to study the function of layer 5 projections to subcortical areas including thalamic nuclei. Previous studies have revealed that *Snap25* cKO mice have memory defects, hyperactivity, and alteration of sleep patterns (Gustus et al. 2018; Hoerder-Suabedissen et al. 2019; Krone et al. 2020). However, *Rbp4-Cre* is expressed not only in layer 5b but also in layer 5a whose neurons have callosal (corticocortical) projections, and it is also expressed in various structures including dentate gyrus in hippocampus (Hoerder-Suabedissen et al. 2019). Those made it difficult to interpret the behavioral phenotypes of *Snap25* cKO mice. Our previous study has also shown that layer 5 axons start to degenerate after P21 and degenerating synapses have dark cytoplasm and multivesicular bodies in the cortex (Hoerder-Suabedissen et al. 2019). Such degenerative aspects were not detected in layer 5-Po synapses at P18. Moreover, the number of microglia was not changed in Po in *Snap25* cKO mice during development. This argues against, but does not rule out, the possibility that degeneration causes the defects in the development of the complex synapses. Conversely, it is possible that the developmental failure found in the present study could eventually lead to later degeneration. To separate developmental defects and degeneration, it is necessary to manipulate the timing of *Snap25* expression more precisely using tools such as the Tet-On/Off system.

Our results clearly indicate that activity mediated through regulated vesicular release from the presynaptic terminal is not necessary for the formation of synapse, but it is required for the establishment of glomerular structures between layer 5 corticothalamic projections in Po. While the previous publications that reported no change in synapse formation used organotypic slice cultures of double knockout of *Munc13-1* and *13-2* (Sigler et al. 2017) or Emx1-driven TeNT-expressing brains (Sando et al. 2017), we used conditional *Snap25* KO in a specialized synapse in vivo. The lack of regulated vesicular release had an obvious effect, but it is not clear how SNAP25-dependent synaptic vesicular release regulates morphological changes of layer 5 boutons and excrescence formation in Po. SNAP25 is involved in the release of neurotransmitters during synaptic transmission and also the secretion of neurotrophic factors such as BDNF (Südhof 2013; Shimojo et al. 2015). Since *Snap25* knockout can affect both processes, *Snap25* cKO alone does not allow us to elucidate the relative contribution of these mechanisms in the formation of layer 5-Po synapses. We would therefore need to employ additional silencing methods or knocking out of factors specific to neurotransmitter release or secretion to compare their effects with those of *Snap25* cKO. The specialized synapses between layer 5 and Po dendrites present excellent model systems to further investigate the molecular mechanisms underlying SNAP25-dependent specialized synaptic development of layer 5 corticothalamic projections in Po.

## Notes

S.H. and Z.M. conceived experiments. S.H., A.H.-S., G.K., and Z.M. wrote the manuscript. S.H. performed histological and ultrastructural analysis of boutons. A.H.-S. performed viral injections and helped supervise the project. C.M. and G.K. carried out the SBEM sample preparation and imagining. E.K. performed pre-embedding immunoelectron microscopy for analysis of synapses with advice from K.T. We thank T. Kaneko for the anti-RFP antibody. *Conflict of interest:* There is no conflict of interest.

## Funding

Medical Research Council (G00900901 to ZM’s laboratory). Daiichi Sankyo Foundation of Life Science, The Uehara Memorial Foundation and KAKENHI (Grant-in-Aid for Research Activity Start-up, 19K23786 to S.H.).

## Supplementary Material

Hayashi_et_al_Combined_Suppl_materials_revised_bhaa379

Suppl_Video1_bhaa379

Suppl_Video2_bhaa379
