# Supplementary material for: Maturation of Complex Synaptic Connections of Layer 5 Cortical Axons in the Posterior Thalamic Nucleus Requires SNAP25
Source: Cereb Cortex. 2020 Dec 26;31(5):2625–38. doi: 10.1093/cercor/bhaa379 (PMC8023812; doi:10.1093/cercor/bhaa379)
Supplement: Hayashi_et_al_Combined_Suppl_materials_revised_bhaa379 [file hayashi_et_al_combined_suppl_materials_revised_bhaa379.docx]

**Supplementary Materials**

**Supplementary Figure 1.**

**
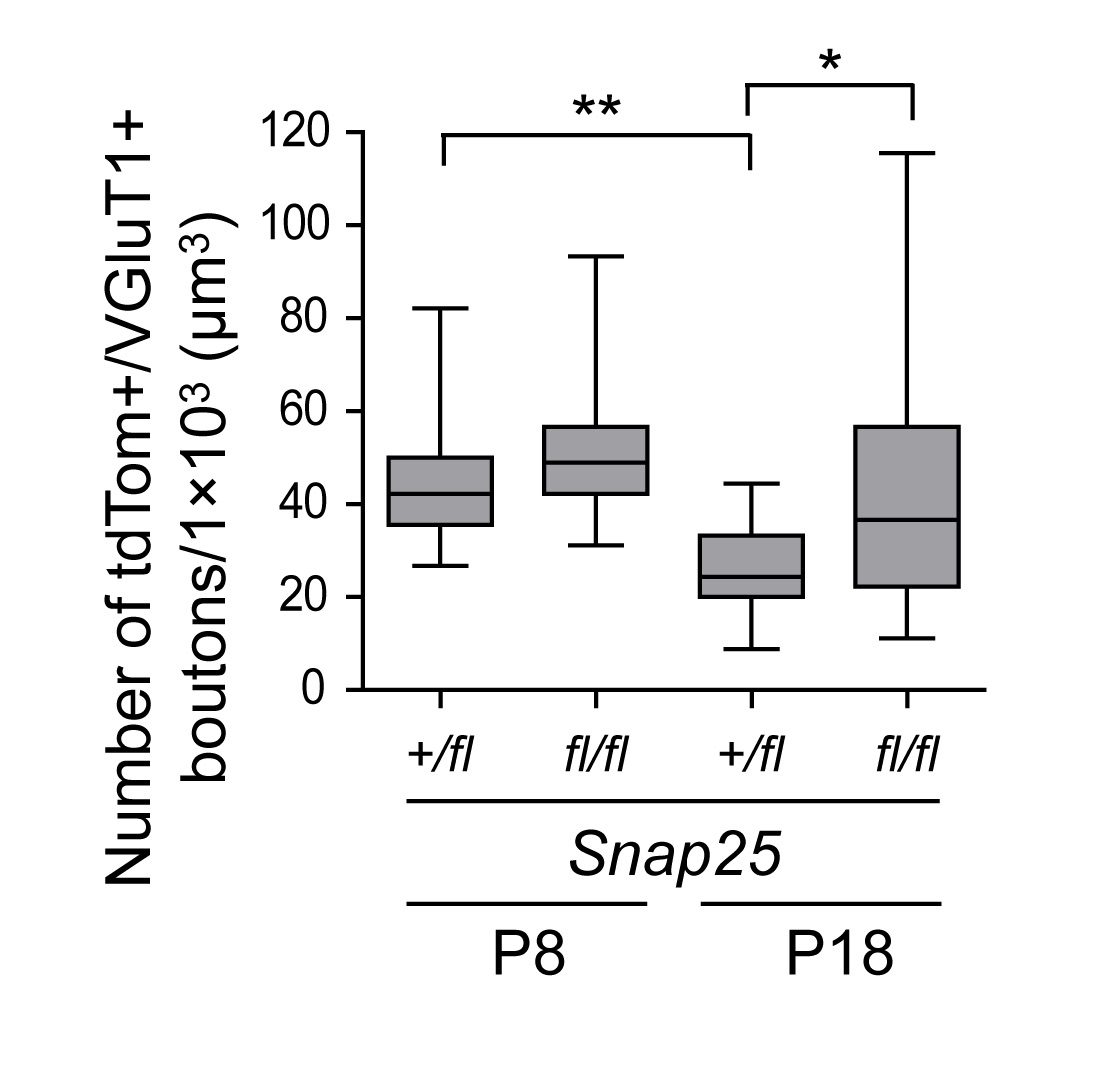
**

**Supplementary Figure 1.** Comparisons of densities of tdTom+ boutons in Po of *Rbp4-Cre;Snap25^+/fl^* and *Rbp4-Cre;Snap25^fl/fl^* at P8 and P18.

The number of tdTom+ and VGluT1+ boutons per 1×10^3^ μm^3^ (density) in Po was not significantly different between *Snap25^+/f^* and *Snap25^fl/fl^* at P8. The density was significantly decreased in P18 compared to P8 in *Snap25^+/f^*. It was more variable in *Snap25^f/f^* at P18 than that of the same genotype at P8 and was significantly larger than that of *Snap25^+/f^* at P18. n=17 and 20 areas from three brains were analysed for both genotypes at P8 and P18, respectively. One-way ANOVA followed by the post hoc test with Dunn's Multiple Comparison Test, *, *P*<0.05; **, *P*<0.01.

**Supplementary Figure 2.**

**
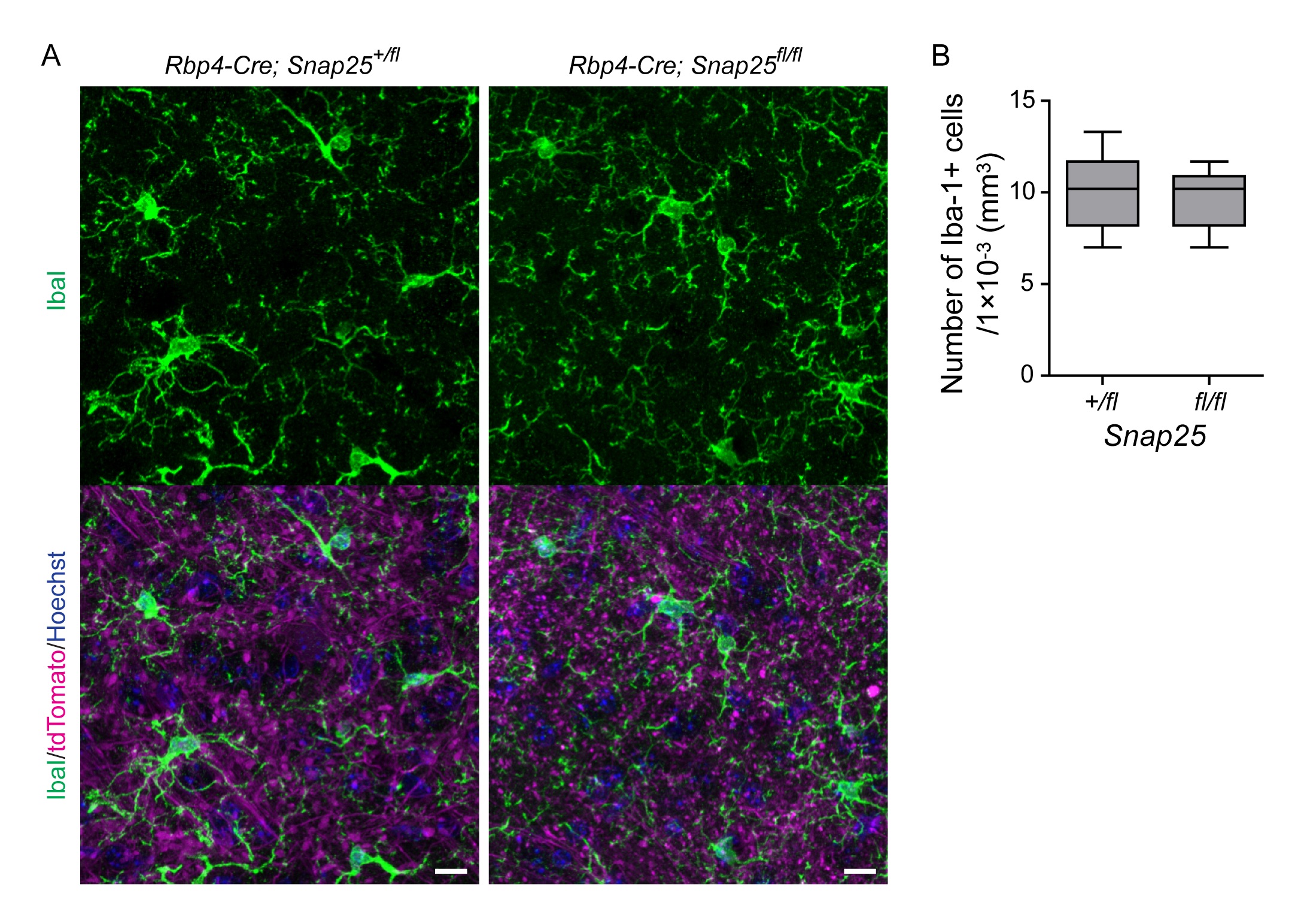
**

**Supplementary Figure 2.** Microglia in Po of *Rbp4-Cre;Snap25^+/fl^* and *Rbp4-Cre;Snap25^fl/fl^* at P21.

**A**, Laser-scanning confocal microscopy images of Iba-1 immunoreactivity (upper) and merged images of Iba-1, tdTomato and Hoechst staining (lower). Maximum intensity projections of z-stack images (total 25 μm-thickness) are shown. tdTomato and Hoechst signals show *Rbp4-Cre*+ axons and nuclei, respectively. **B**, The number of Iba-1-positive cells per 1×10^-3^ mm^3^ in Po. n= 18 areas were analysed from three brains for each genotype. There was no significant difference between the two. Mann-Whitney U Test. Scale bar, 10 μm.

**Supplementary Figure 3.**

**
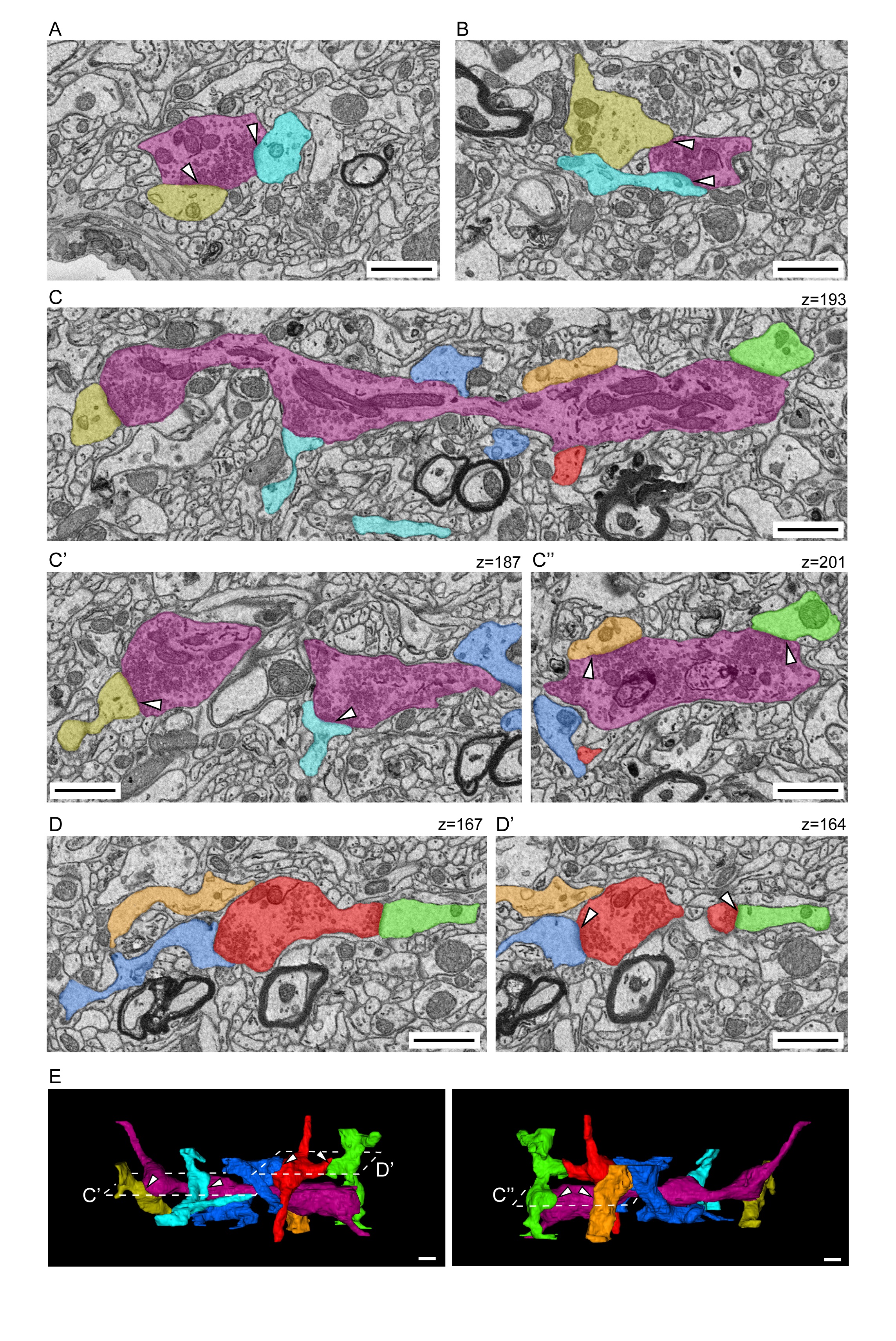
**

**Supplementary Figure 3.** Examples for *Rbp4-Cre;Snap25 ^fl/fl^* boutons that form more than one synapse with different dendrites in Po at P18.

**A-D**, Four examples of axons in Po of *Rbp4-Cre;Snap25 ^fl/fl^* brains that form two synapses within single boutons (**A,B**) or form multiple boutons within a short distance (~10 μm) (**C,D**). Image from a single section selected from the series (plane shown in E) taken with the SBEM are shown. Magenta (**A**- **C**) and red (**C**,**D**) indicate axons, and other colours (blue, green, beige) indicate dendrites. **C’**, **C’’** and **D’** show synapses formed at different z-levels of the axons shown in **C** (magenta) and **D** (red), respectively. Red in **C** and **D** depicts the same axon that forms synapses at the level (z=164) shown in **D’**. **E**, 3D model of reconstructed axons and dendrites shown in **C** and **D**  (top, z=116; bottom, z=238). Left and right images show front and back view of the reconstructed model, respectively. Scale bar, 1 μm.

**Supplementary Video 1**

Animation of ultrastructural image series taken with the SBEM from Po in an *Rbp4-Cre;Snap25^+/+^* brain. Scale bar, 1 μm.

**Supplementary Video 2**

Animation of ultrastructural image series taken with the SBEM from Po in an *Rbp4-Cre;Snap25 ^fl/fl^* brain. Scale bar, 1 μm.
